# Efficient embedding of complex networks to hyperbolic space via their Laplacian

**DOI:** 10.1038/srep30108

**Published:** 2016-07-22

**Authors:** Gregorio Alanis-Lobato, Pablo Mier, Miguel A. Andrade-Navarro

**Affiliations:** 1 Faculty of Biology, Johannes Gutenberg Universität, Institute of Molecular Biology, Ackermannweg 4, 55128 Mainz, Germany

## Abstract

The different factors involved in the growth process of complex networks imprint valuable information in their observable topologies. How to exploit this information to accurately predict structural network changes is the subject of active research. A recent model of network growth sustains that the emergence of properties common to most complex systems is the result of certain trade-offs between node birth-time and similarity. This model has a geometric interpretation in hyperbolic space, where distances between nodes abstract this optimisation process. Current methods for network hyperbolic embedding search for node coordinates that maximise the likelihood that the network was produced by the afore-mentioned model. Here, a different strategy is followed in the form of the Laplacian-based Network Embedding, a simple yet accurate, efficient and data driven manifold learning approach, which allows for the quick geometric analysis of big networks. Comparisons against existing embedding and prediction techniques highlight its applicability to network evolution and link prediction.

The gradual addition of nodes and edges to a network, a common representation of the relationships between complex system components, imprints valuable information in its topology. Consequently, development of techniques to mine the structure of networks is crucial to understand the factors that play a role in the formation of their observable architecture.

Strong clustering and scale-free node degree distribution, properties common to most complex networks, have served as the basis for the establishment of tools for link prediction^1^, community detection^2^, identification of salient nodes^3^, and so on. In addition, several models that aim to mimic the evolution and formation of networks with the above-mentioned characteristics have been introduced^4^. Of special interest are a series of models that assume the existence of a hidden geometry underlying the structure of a network, shaping its topology^5^,^6^,^7^,^8^,^9^,^10^,^11^,^12^ (we refer the reader to^13^ for an extensive review on the subject). This is justified by the fact that complex networks possess characteristics commonly present in geometric objects, like scale invariance and self-similarity^7^,^14^,^15^,^16^.

One of such models is the so-called Popularity-Similarity (PS) model, which sustains that clustering and hierarchy are the result of an optimisation process involving two measures of attractiveness: node popularity and similarity between nodes^12^. Popularity reflects the property of a node to attract connections from other nodes over time, and it is thus associated with a node’s seniority status in the system. On the other hand, nodes that are similar have a high likelihood of getting connected, regardless of their rank.

The PS model has a geometric interpretation in hyperbolic space, where the trade-off between popularity and similarity is abstracted by the hyperbolic distance between nodes^9^,^12^. Short hyperbolic distances between them correlate strikingly well with high probabilities of link formation^12^. This means that mapping a network to hyperbolic space unveils the value of the variables in charge of shaping its topology (popularity and similarity in this case), allowing for a better understanding of the dynamics accountable for the system’s growth process.

Current efforts to infer the hyperbolic geometry of complex networks bet for a Maximum Likelihood Estimation (MLE) approach, in which the space of PS models with the same structural properties as the network of interest is explored, in search for the one that better fits the network topology^12^,^17^,^18^. This search is computationally demanding, which means that these methods require of correction steps or heuristics in order to make them suitable for big networks^18^.

In this paper, a different strategy is followed. Inspired by the well-established field of non-linear dimensionality reduction in Machine Learning^19^, an adaptation of the Laplacian Eigenmaps algorithm, introduced by Belkin and Niyogi for the low-dimensional representation of complex data^20^, is put forward for the embedding of complex networks into the two-dimensional hyperbolic plane. The proposed approach is based on the approximate eigen-decomposition of the network’s Laplacian matrix, which makes it quite simple yet accurate and efficient, allowing for the analysis of big networks in a matter of seconds. Furthermore, it is data driven, which means that no assumptions are made about the model that constructed the network of interest. Finally, benchmarking of the method against existing embedding and prediction techniques highlights the advantages of using it for the prediction of new links between nodes and for the study of network evolution.

## Results

### Preliminaries and the proposed method

In this paper, only undirected, unweighted, single-component networks are considered, as the proposed embedding approach is only applicable to networks with these properties^20^. Moreover, these networks are assumed to be scale-free (with scaling exponent *γ* ∈ [2, 3]) and with clustering coefficient 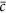 significantly larger than expected by chance. These networks are graphs *G* = (*V*, *E*) with *N* = |*V*| nodes and *L* = |*E*| edges connecting them. An undirected, unweighted graph can be represented by an *N* × *N* adjacency matrix *A*_*i*,*j*_ = *A*_*j*,*i*_ ∀*i*, *j*, whose entries are 1 if there is an edge between nodes *i* and *j* and 0 otherwise. The graph Laplacian is a transformation of *A* given by *L* = *D* − *A*, where *D* is a matrix with the node degrees on its diagonal and 0 elsewhere.

**Figure 1:**
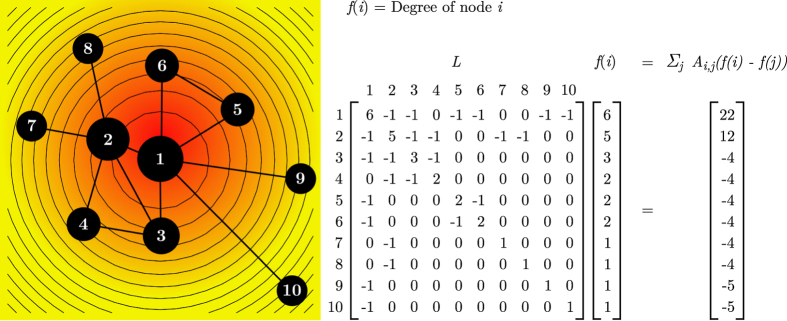
The graph Laplacian. A three-dimensional surface is depicted by a contour plot overlaid on a heat map. The surface is approximated by a network, whose nodes have more edges where the value of its Laplacian is greater (red region of the heat map). Let a function 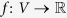 act on each of the *N* nodes and produce its degree. The network’s Laplacian, a discrete version of the one acting on the surface, can be used as an operator on *f* (*i*), giving information about how the degree of each node *i* compares to that of its neighbours *j*, i.e. ∑_*j*_ *A*_*i*, *j*_(*f* (*i*) – *f* (*j*)). Node size is proportional to node degree. The numbers labelling each node indicate their row/column in the Laplacian matrix.

Let us now consider a real-valued function on the set of network nodes, 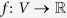, which assigns a real number *f* (*i*) to each graph node. If the Laplacian acts as an operator over this function, *Lf* (*i*) = ∑_*j*_*A*_*i*,*j*_(*f* (*i*) − *f* (*j*)), one can see that it is giving information about how the value of *f* for each node *i* compares to that of its neighbours *j*^21^. This is the discrete analogue of the Laplace operator in vector calculus and its generalisation in differential geometry, the Laplace-Beltrami operator^20^, which measure how much the curvature of a surface is changing at a given point. This is more evident if one thinks of a function as being approximated by a graph, such that nodes have more edges where the value of the Laplacian is greater (see Fig. 1).

In particular, embedding of the network to the two-dimensional hyperbolic plane 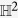, represented by the interior of a Euclidean circle^9^, is given by the *N* × 2 matrix *Y* = [**y**_**1**_, **y**_**2**_] where the *i*th row, *Y*_*i*_, provides the embedding coordinates of node *i*. Using the Laplacian operator (see above), this corresponds to minimising 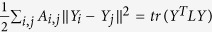, which reduces to 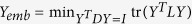 with *D* as defined above, *I* the identity matrix, *M*^*T*^ the transpose of *M* and *tr*(*M*) the trace of *M*. Finally, *Y*_*emb*_, the matrix that minimises this objective function, is formed by the two eigenvectors with smallest non-zero eigenvalues that solve the generalised eigenvalue problem *LY* = *λDY*^20^ (see Section 1 in the Supplementary Information for a detailed justification of this embedding approach).

**Figure 2:**
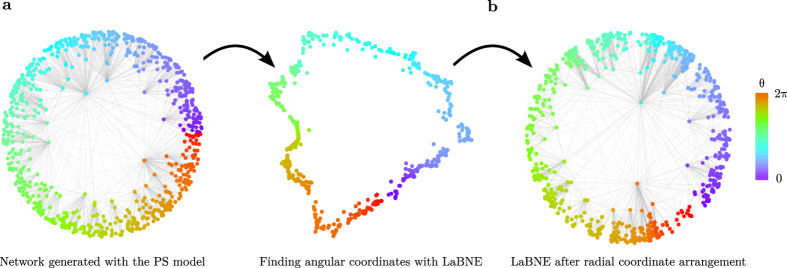
Laplacian-based Network Embedding. **(a)** A network of 750 nodes was generated by means of the PS model, with target average node degree 2*m* = 10, scaling exponent *γ* = 2.75 and network temperature *T* = 0. The network is embedded to the hyperbolic plane 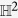 with LaBNE to reveal the angular position of the nodes in the hyperbolic circle containing the network. **(b)** Finally, the radial coordinates of the nodes are assigned, so that they resemble the rank of each node according to its degree. By the colour of the nodes, which highlights their angular coordinates, one can note that the embedding by LaBNE is rotated by some degrees with respect to the actual node angular coordinates obtained with the PS model. This does not impact the hyperbolic, distance-dependent connection probabilities, because distances are invariant under rotations. Edges in the raw embedding by LaBNE are not shown for clarity.

In the context of manifold learning, most algorithms rely on the construction of a mesh or network over the high-dimensional manifold containing the samples of interest^19^,^22^. When pairwise distances between samples are computed, they correspond to shortest-paths over the constructed network, allowing for a better preservation of the sample relationships when the data is embedded to low dimensions^19^,^20^,^22^,^23^. If there is really a hyperbolic geometry underlying a complex network, it should lie on a hyperbolic plane, with nodes drifting away from the space origin. If the network itself is seen as the mesh that connects samples (nodes in this case) that are close to each other^12^, it can be used as in manifold learning to recover the hyperbolic coordinates of its nodes. Connected pairs of nodes in the network should be very close to each other in the target, low-dimensional space (hence the minimisation problem presented above) and, consequently, their angular separation (governed by their similarity dimension according to the PS model) should also be small. Figure 2a shows that, if the described embedding approach is employed, this is indeed the case for an artificial network generated with the PS model (see the Methods for more details).

As a result, to complete the mapping to 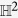, angular node coordinates are obtained via ***θ*** = arctan(**y**_**2**_/**y**_**1**_) and radial coordinates (abstracted by the popularity or seniority dimension in the PS model) are chosen so as to resemble the rank of each node according to its degree. This is achieved via *r*_*i*_ = 2*β* ln(*i*) + 2(1 − *β*) ln(*N*), where nodes *i* = {1, 2, …, *N*} are the network nodes sorted decreasingly by degree and *β* = 1/(*γ* − 1)^9^,^12^ (see Fig. 2b and the Methods for further details).

This strategy is valid, because the native representation of 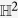, in which the hyperbolic space is contained in a Euclidean disc and Euclidean and hyperbolic distances from the origin are equivalent, is a conformal model. This means that Euclidean angular separations between nodes are also equivalent to hyperbolic ones^9^. On the other hand, the radial arrangement of nodes corresponds to a quasi-uniform distribution of radial coordinates in the disc^9^. It is also important to mention that, due to rotational invariance of distances, the set of hyperbolic coordinates responsible for the edges observed in a network is not unique (see Fig. 2b). Therefore, the goal of the proposed technique is not to find a specific set of coordinates, but the one that corresponds better with the hyperbolic, distance-dependent connection probabilities that produce the network of interest.

The network embedding approach described in this section and in Table 1 is hereafter referred to as Laplacian-based Network Embedding (LaBNE).

### Benchmarking LaBNE

In order to test the ability of LaBNE to infer the hyperbolic geometry of complex networks, artificial networks were generated using the PS model. This model allows for the construction of networks with known hyperbolic coordinates for each of its *N* nodes and target average node degree 2*m*, scaling exponent *γ* and clustering coefficient 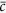. The latter is controlled by the network temperature *T*, is reduced almost linearly with *T* and is the strongest possible at *T* = 0^9^ (see Methods for more details). One hundred synthetic networks were grown for each combination of parameters *T* = {0, 0.3, 0.6, 0.9}, 2*m* = {4, 6, 8, 10} and *γ* = {2.25, 2.50, 2.75}; the number of nodes *N* was fixed to 500. These networks were then mapped to 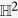 with LaBNE, and Pearson correlations between the inferred hyperbolic distances and the ones measured with the real node coordinates were computed and averaged across the one hundred networks for each parameter configuration. This same procedure was followed for the most recent and fastest version of HyperMap, a MLE method for network embedding to hyperbolic space that finds node coordinates by maximising the likelihood that the network is produced by the PS model^17^,^18^ (see Methods for details).

**Figure 3:**
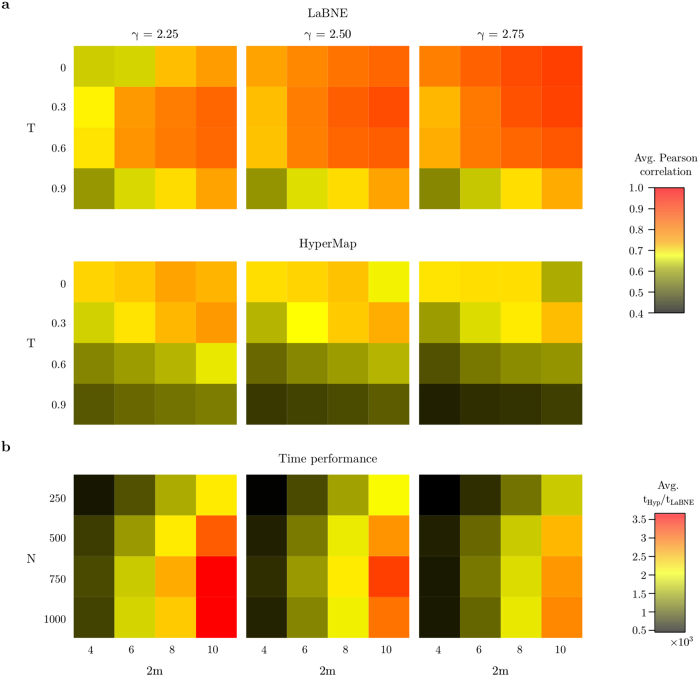
Benchmarking LaBNE. **(a)** For each combination of the depicted parameters *N* = 500, *γ*, *T* and 2*m*, 100 artificial networks were generated with the PS model. Each network was embedded to the hyperbolic plane with LaBNE and the most recent and fastest version of HyperMap, and the Pearson correlation between the real node distances and the inferred ones was computed. The average Pearson correlation across the 100 networks is shown for each parameter combination. **(b)** The average time required by HyperMap and LaBNE to embed each of the 100 artificial networks per parameter configuration was compared via the fold-change from the latter to the former. In this case, *T* was fixed to 0 and *N* changed from 250 to 1000.

Figure 3a shows how the average correlation for each parameter combination, obtained by LaBNE, is as high or higher than the one obtained by HyperMap. This is especially evident in dense, strongly clustered networks (i.e. networks with large 2*m* and *T* → 0, which implies high 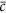). These results are supported by the fact that angular coordinates inferred by LaBNE are closer to the angles from the generated PS networks, compared to the ones inferred by HyperMap (see Figure S1).

To provide an in-depth analysis of the differences in time performance of the two algorithms, an experiment similar to the one presented in Fig. 3a was carried out, but this time parameter *T* was fixed to 0 and the network size changed as *N* = {250, 500, 750, 1000}. Figure 3b depicts that the fold-change from the average time needed by LaBNE to embed each of the one hundred networks per parameter configuration to the average time needed by HyperMap to perform the same task is very big, the latter being 500 times slower than LaBNE for the smallest networks. These results highlight one of the great benefits of LaBNE, its time performance, which stems from its computational complexity of *O*(*kN*^2^) with *k* = 3. In a network with a single connected component, the smallest eigenvalue of *L* is always 0^21^ and its corresponding eigenvector is discarded. The next two, which correspond to the smallest non-zero eigenvalues, form the resulting node coordinates. Since *k* is always constant, LaBNE can be considered a *O*(*N*^2^) algorithm.

In spite of the version of HyperMap considered here being a *O*(*N*^2^) method as well^18^ (see Methods for details), Fig. 3b shows that it is slower than LaBNE. This is because the heuristic used to speed up this algorithm is not actually applied to all *N* nodes in the network of interest, but only to those with degrees smaller than a parameter *k*_*speedup*_^18^ (see Methods for details). The larger the value of this parameter, the faster the algorithm, but also the higher the impact on its accuracy. It was set to 10 throughout this paper, which produced good results in a reasonable amount of time. On the other hand, LaBNE takes advantage of the efficiency and portability of the R package igraph^24^ and its interface with high-performance subroutines designed to solve large scale eigenvalue problems^25^.

### Hyperbolic embedding for link prediction in real networks

Given the accuracy and time performance achieved by LaBNE, and considering that short hyperbolic distances correlate well with high probabilities of connection between nodes^12^, LaBNE was used to infer the hyperbolic coordinates for the nodes of three real networks (see Table 2 and Methods) to then carry out link prediction. As discussed in the previous section, Figure 3 shows that the accuracy of LaBNE is higher for networks with more topological information, i.e. with more edges between nodes, which occurs at high average node degrees (2*m*) and low temperatures (which imply high clustering 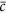). Consequently, the three real networks analysed here were chosen with the aim to investigate the performance of LaBNE in the low, medium and high clustering coefficient scenarios (see Table 2). Furthermore, these network datasets represent complex systems from different domains: the high quality human protein interaction network (PIN) models the relationships between proteins within the human cell (low 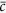), in the Pretty-Good-Privacy network (PGP) users share encryption keys with people they trust (medium 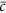) and the autonomous systems Internet (ASI) corresponds to the communication network between groups of routers (high 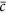, see the Methods for more details).

Topological link prediction deals with the task of predicting links that are not present in an observable network, based merely on its structure. The standard way to evaluate the performance of a link predictor is to randomly remove a certain number of links from the network under study, use a predictor to assign likelihood scores to all non-adjacent node pairs in the pruned topology, sort the candidate links from best to worst based on their scores, to finally scan this sorted list of candidates with a moving threshold to compute Precision (fraction of candidate links that pass the current score threshold and are in the set of removed links) and Recall (fraction of candidate links that have not passed the score threshold but are in the set of removed links) statistics^1^,^26^.

The above-described evaluation framework comes, however, with a critical caveat. Pruning edges at random can remove important information from the observable network topology in unpredictable ways, and this most certainly affects link predictors differently^26^. To avoid this problem, historical data of the evolution of the network must be used to test the ability of a link predictor to assign high likelihood scores to edges in a network *G*_*t* + 1_ that are not yet present in a snapshot *G*_*t*_, to which the predictor is applied. For the ASI^27^ and the PGP, temporal snapshots of their topology were available and this evaluation method was used (see networks with subscript *t* + 1 in Table 2). For the PIN^28^, it was necessary to resort to the so-called Guilt-by-association Principle^29^, which states that two proteins are highly likely to interact if they are involved in the same biological process. In this scenario, link predictors are applied to the observable protein network and discrimination between good and bad candidate interactions is based on a stringent cut-off on a measure of the similarity between the biological processes of the non-adjacent proteins (see the Methods for more details).

**Figure 4:**
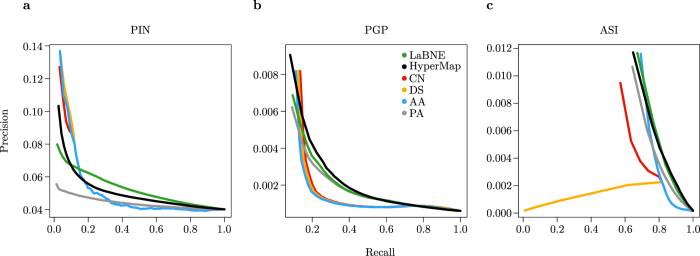
Link prediction in real networks. Precision-Recall curves for the three networks analysed: **(a)** the high quality protein interaction network (PIN), **(b)** the Pretty-Good-Privacy web of trust (PGP) and **(c)** the autonomous systems Internet (ASI). Note that despite LaBNE being an approximate method, its performance is similar to HyperMap’s, especially on strongly clustered networks like the ASI. The performance of other topological link predictors - the Common Neighbours index (CN), the Dice Similarity (DS), the Adamic and Adar index (AA) and the Preferential Attachment index (PA) - is also shown. Some curves start late in these plots, because the first set of best candidate links considered produced high Recall levels right away.

Figure 4a–c shows the performance in link prediction of LaBNE, HyperMap and a set of neighbourhood-based link predictors that are commonly considered for benchmarking in this context (see Methods). As expected from the results on artificial networks (Fig. 3a), the performance of both LaBNE and HyperMap improves as clustering increases. The worst results are thus obtained in the PIN (Fig. 4a). However, LaBNE is the only prediction technique that allows for more Recall without sacrificing as much Precision as the others. The performance of HyperMap in this network is bad because, as it can be seen in Fig. 3a and S1b, its application should be restricted to highly clustered networks. In the other two cases (Fig. 4b,c), LaBNE is the second best performing method in terms of area under the Precision-Recall curve, only slightly behind HyperMap. These are very good results considering that LaBNE can obtain reliable link predictions in these big networks in a matter of seconds, while the current implementation of the most recent and fastest version of HyperMap requires days to produce the results (see Table 2).

Regarding the performance of the neighbourhood-based link predictors, it is important to highlight their good Precision at low Recall levels. This is mainly due to the fact that they are only able to assign meaningful scores to node pairs separated by at most two hops, which is a very small fraction of all possible candidate edges. The rest obtain the exact same score, which prevents from differentiating between good and bad candidate interactions and results in the rapid drop of Precision observed in Fig. 4a–c.

### Hyperbolic embedding for real network evolution analysis

In the PS model, radial coordinates are directly proportional to node birth-times, i.e. if a node *i* is close to the origin of the hyperbolic circle containing the network (*r*_*i*_ → 0), it means it was born early in the evolution of the complex system^12^. To test whether this is the case in the most recent temporal snapshot of the three real networks considered (PIN, PGP and ASI), node radial coordinates inferred by LaBNE were compared to actual node birth-times (see the Methods for details on how birth-times are defined for each real network; HyperMap was not used here as it practically produces the same radial coordinates as LaBNE).

**Figure 5:**
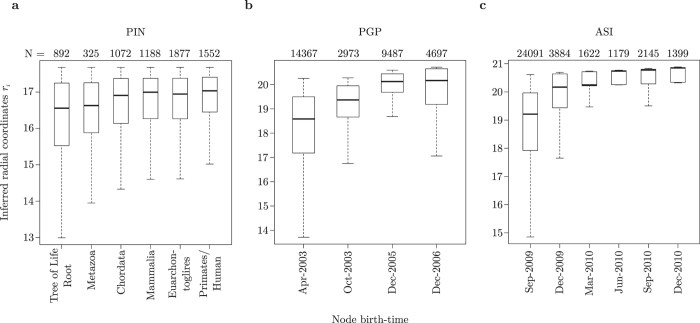
Inferred radial coordinates resemble actual node birth-times. Senior nodes of the three networks analysed - **(a)** the high quality human protein interaction network (PIN), **(b)** the Pretty-Good-Privacy web of trust (PGP) and **(c)** the autonomous systems Internet (ASI) - have radial coordinates that are close to the centre of the hyperbolic disc containing the network, as opposed to younger nodes, which are placed in the circle’s periphery. The numbers above each box-plot indicate the number of network nodes that were assigned to the corresponding birth-time.

Figure 5a–c shows that in the three cases, nodes that are close to the centre of the hyperbolic space are older than those located in its periphery. Even all nodes from the first network snapshot in the PGP and ASI, which represent a mix of nodes that appeared at that time and nodes from older, not available time-points, possess small radial coordinates (Fig. 5b,c).

These are very important results, because they exhibit the close relationship between node popularity and seniority in networks of very different objects and time scales. The results obtained with LaBNE suggest that, even when the identity of the network nodes is unknown, one can have an idea of their history in the system under study, based merely on their degree and, consequently, their inferred radial positions.

## Conclusions

Scale-invariance, self-similarity and strong clustering, properties present in complex systems and geometric objects alike, have led to the proposal that the network representations of the former lie on a geometric space where distance constraints play important roles in the formation of links between system components^8^,^9^,^12^,^30^. One of such proposals advocates for the hyperbolic space as a good candidate to host complex networks, given that their skeletons (trees abstracting their underlying hierarchical structure) require an exponential space to branch and only hyperbolic spaces expand exponentially^9^.

In consequence, efficient and accurate methods to embed networks to hyperbolic space are needed. In this article, a novel approach to perform this task is proposed: the Laplacian-based Network Embedding or LaBNE. Since it is based on a transformation of the adjacency matrix representation of a network, namely the graph Laplacian, it highly depends on topological information to carry out good embeddings. This was confirmed when applied to artificial and real networks with differing structural characteristics. The higher the average node degree (2*m*) and clustering coefficient (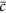) of a network, the better the results achieved by LaBNE. Nevertheless, its low computational complexity allows for the study of the hyperbolic geometry of big networks in a matter of seconds. This means that LaBNE is suited to draft a geometric configuration of a network, which can then be used by more involved and time consuming techniques, thus reducing the space of possible node coordinates they have to explore.

Notwithstanding the fact that techniques for embedding networks to generic low-dimensional spaces have been proposed to facilitate their visualisation and analysis^19^,^20^,^22^,^23^,^30^,^31^,^32^,^33^, it is important to stress that LaBNE deals specifically with the embedding to the two-dimensional hyperbolic plane. This space has been shown to provide an accurate reflection of the geometry of real networks^9^,^12^ (see Figs 4 and 5) and allows for their visual inspection in two or three dimensions (see Figure S2). However, LaBNE does not make any *a priori* assumption about the model or mechanism that led to the formation of the network of interest. Thus, the distance-dependent connection probabilities resulting from the mapping to 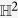 serve as the basis to determine if such a space is suitable for the network or not. For example, networks grown with the Barabasi-Albert model^14^ are infinite-dimensional hyperbolic networks^34^, but short distances between their nodes, measured with the coordinates inferred by LaBNE or HyperMap in 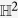, are not indicative of link formation in this space (see Figure S3). The in-depth study of networks with high-dimensional latent spaces or their embedding to 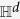 (with *d* > 2) are of great interest, but beyond the scope of this article.

Finally, although this work did not intend to provide an extensive comparison between link predictors or a thorough analysis of the evolution of real networks, it is important to note that LaBNE performed very well in these two type of studies when applied to a biological, a social and a technological network. These represent a few example scenarios in which the inference of the hyperbolic geometry underlying a network could be useful.

## Methods

### The PS model

The PS model^12^ on the hyperbolic plane of curvature *K* = −1 is formulated as follows: (1) initially the network is empty; (2) at time *t* ≥ 1, a new node *t* appears at coordinates (*r*_*t*_, *θ*_*t*_) with *r*_*t*_ = 2 ln*t* and *θ*_*t*_ uniformly distributed on [0, 2*π*], and every existing node *s* < *t* increases its radial coordinate according to *r*_*s*_(*t*) = *β r*_*s*_ + (1 − *β*) *r*_*t*_ with *β* = 1/(*γ* − 1) ∈ [0, 1]; (3) new node *t* picks a randomly chosen node *s* < *t* that is not already connected to it and links with it with probability 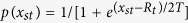, where parameter *T*, the network temperature, controls the network’s clustering coefficient, 
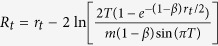
 is the current radius of the hyperbolic circle containing the network, *x*_*st*_ = *r*_*s*_ + *r*_*t*_ + 2ln(*θ*_*st*_/2) is the hyperbolic distance between nodes *s* and *t* and *θ*_*st*_ is the angle between the nodes; (4) repeat step 3 until node *t* gets connected to *m* different nodes; (5) repeat steps 1–4 until the network is comprised of *N* nodes. Note that if *T* → 0, 
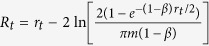
. In addition, if *β* = 1/(*γ* − 1) = 1, existing nodes do not change their radial coordinates and *R*_*t*_ = 2ln*t*.

### Radial arrangement of nodes in LaBNE

As described above, new nodes in the PS model acquire radial coordinates *r*_*t*_ = 2ln*t* that depend on their birth-time *t*. This means that the probability of finding a node that is close to the centre of the hyperbolic circle containing the growing network, is exponentially lower than the probability to find a node on the periphery. When a new node is added to the system and the existing ones change their radial position according to *r*_*s*_(*t*) = *β r*_*s*_ + (1 − *β*) *r*_*t*_, where *β* = 1/(*γ* − 1), their seniority is attenuated by increasing their distances to every newly added node^12^. Consequently, the *N* angular coordinates found by LaBNE are complemented with the nodes’ radial coordinates obtained via *r*_*i*_ = 2*β* ln(*i*) + 2(1 − *β*) ln(*N*), where nodes *i* = {1, 2, …, *N*} are the network nodes sorted decreasingly by degree.

### HyperMap

HyperMap^17^ is a Maximum Likelihood Estimation method to embed a network to hyperbolic space. It finds node coordinates by replaying the network’s hyperbolic growth and, at each step, maximising the likelihood that it was produced by the PS model^17^. For embedding to the hyperbolic plane of curvature *K* = −1 it works as follows: (1) nodes are sorted decreasingly by degree and labelled *i* = {1, 2, …, *N*} from the top of the sorted list; (2) node *i* = 1 is born and assigned radial coordinate *r*_1_ = 0 and a random angular coordinate *θ*_1_ ∈ [0, 2*π*]; (3) for each node *i* = {2, 3, …, *N*}: (3.1) node *i* is born and assigned radial coordinate *r*_*i*_ = 2ln*i*; (3.2) the radial coordinate of every existing node *j* < *i* is increased according to *r*_*j*_(*i*) = *β r*_*j*_ + (1 − *β*) *r*_*i*_; (3.3) node *i* is assigned the angular coordinate *θ*_*i*_ maximising the likelihood 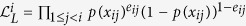. *β* and *p*(*x*_*ij*_) are defined as in the PS model and *e*_*ij*_ is 1 if nodes *i* and *j* are connected and 0 otherwise. The maximisation of 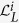 is performed numerically by trying different values of *θ* in [0, 2*π*], separated by intervals Δ*θ* = 1/*i*, and then choosing the one that produces the greatest 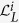.

Since the angular coordinates yielded by this link-based likelihood are not very accurate for small *i* (i.e. for high degree nodes)^17^, the fast version of HyperMap used in this paper uses information on the final number of common neighbours between these old nodes via the maximisation of the log-likelihood 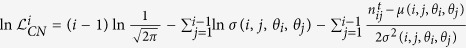, where *μ* is the mean number of common neighbours *n*_*ij*_ between *i* and *j* and *σ*^2^ is the associated variance^18^. This hybrid version of HyperMap is *O*(*N*^3^) and to speed it up, Papadopoulos and colleagues resort to the following heuristic: for nodes *i* with degree *k*_*i*_ < *k*_*speedup*_, an initial estimate 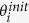 of their angular coordinate is computed by considering only the previous nodes *j* < *i* that are their neighbours; these estimates are then refined, searching for the final *θ*_*i*_ within a small region around 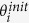. The fast hybrid version of HyperMap with *k*_*speedup*_ = 10 is the one used throughout this work. We refer the reader to^18^ for more details on the speed-up heuristic and the derivation of 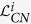. Finally, even when correction steps can be used together with the fast hybrid HyperMap, their effect on this method has been reported not to be significant^18^ and they are not considered here.

### Network datasets

For the three network datasets used in this paper, self-loops and multiple edges were discarded and only the largest connected component was considered.

The high-quality protein interaction network (PIN) is a stringent subset of the Human Integrated Protein-Protein Interaction rEference (HIPPIE)^28^. HIPPIE retrieves interactions between human proteins from major expert-curated databases and calculates a score for each one, reflecting its combined experimental evidence. This score is a function of the number of studies supporting the interaction, the quality of the experimental techniques used to measure it and the number of organisms in which the orthologs of the interacting human proteins interact as well. In this paper, only interactions with confidence scores ≥0.73 (the upper quartile of all scores) in release 1.7 were considered. The raw version of this network is available at http://cbdm-01.zdv.uni-mainz.de/ mschaefer/hippie/download.php. To determine the birth-time of the PIN nodes, proteins from the manually curated database SwissProt were clustered based on near full-length similarity and/or high threshold of sequence identity using FastaHerder2^35^. If proteins from two evolutionarily distant organisms are present in one cluster, this suggests that the protein family is ancient. The minimum common taxonomy from all proteins that are part of a cluster was taken as an indication of the cluster’s age. Each node of the PIN was assigned to one of the following age clusters: Tree of Life Root, Metazoa, Chordata, Mammalia, Euarchontoglires or Primates/Human.

Pretty-Good-Privacy (PGP) is a data encryption and decryption program for secure data communication. In a PGP web of trust, each user (node) knows the public key of a group of people he trusts. When user A wants so send information to user B, this information is encrypted with B’s public key and signed with A’s private key. When B receives the information, he verifies that the message is coming from one of the users he trusts and decrypts it with his private key^36^. This encryption and decryption event, forms a directed link between users A and B. In this article, however, the edge directionality of this network is not considered. This is not a problem for the interpretation of the network if we assume that by sharing a key, two users reciprocally endorse their trust in each other^12^. The four temporal snapshots of the undirected PGP network used here, which were collected by Jörgen Cederlöf 37, were used to assign a birth-time for each user based on the snapshot in which he first appeared. The snapshots correspond to April and October 2003, December 2005 and December 2006. The raw PGP data is available at http://www.lysator.liu.se/ jc/wotsap/wots2/.

The autonomous systems Internet (ASI) corresponds to the communication network between IPv4 Internet sub-graphs comprised of routers, as collected by the Center for Applied Internet Data Analysis^27^. The six available network snapshots, spanning the period from September 2009 to December 2010 in 3-month intervals, were used to determine the birth-time of each autonomous system based on the snapshot in which it first appeared. These Internet topologies are available for download at https://bitbucket.org/dk-lab/2015_code_hypermap.

### Link prediction

The performance of LaBNE and HyperMap in link prediction was compared to that of reference neighbourhood-based link predictors. They receive this name because the scores they produce are usually based on how much overlap there is between the neighbourhoods of non-adjacent pairs of nodes in a network. These unlinked nodes are often called *seed nodes*.

The simplest predictor considered was the Common Neighbours (CN) index, which just counts the number of common neighbours between seed nodes^38^. The other indices examined were the Dice Similarity (DS), which is one of the possible normalisations of CN^39^; the Adamic and Adar (AA) index, which assigns higher likelihood scores to seed nodes whose CNs do not interact with other components^40^; and the Preferential Attachment index (PA), which is simply the degree product of the seed nodes^38^. The formulae for these indices are, respectively: *CN*(*x*, *y*) = |Γ(*x*) ∩ Γ(*y*)|, *DS*(*x*, *y*) = 2*CN*(*x*, *y*)/(|Γ(*x*)| + |Γ(*y*)|), 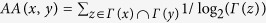 and *PA*(*x*, *y*) = |Γ(*x*)||Γ(*y*)|, where Γ(*x*) is the set of neighbours of *x* and |Γ(*x*)| is the set cardinality.

The embedding- and neighbourhood-based predictors were applied to all the seed nodes of a network snapshot *G*_*t*_, these node pairs were later sorted from best to worst score. This sorted list was scanned with a moving score threshold from top to bottom to compute the proportion of candidate interactions taken that coincide with the set of new edges in *G*_*t*+1_ (Precision) and the proportion of candidate interactions not taken at each threshold but that belong to the set of new edges present in *G*_*t*+1_ (Recall). This allowed for the construction of a Precison-Recall curve for each of the predictors considered.

For the PGP, *G*_*t*_ has 14367 nodes and 37900 edges (snapshot from April 2003), while *G*_*t*+1_ has 31524 nodes and 168559 edges (all the nodes and edges from April 2003 to December 2006). Note, however that only the 62547 new links between the same set of 14367 nodes present in *G*_*t*_ are considered.

For the ASI, *G*_*t*_ has 24091 nodes and 59531 edges (snapshot from September 2009), while *G*_*t*+1_ has 34320 nodes and 128839 edges (all the nodes and edges from September 2009 to December 2010). Note, however that only the 48119 new links between the same set of 24091 nodes present in *G*_*t*_ are considered.

For the PIN, it was necessary to follow a different procedure, as edge timestamps are not available for this network. When the performance of a link predictor is assessed in protein networks, researchers have opted for using Gene Ontology (GO) similarities to discriminate between good and bad candidate interactions^30^,^32^,^41^,^42^. This is based on the Guilt-by-association Principle, which states that if two proteins are involved in similar biological processes, they are more likely to interact^29^. So, the link predictors were applied to the non-adjacent protein pairs in the observable network topology of the PIN and then sorted from best to worst score. The GO similarity of the top 10% candidate links was then computed, together with the proportion of protein pairs with similarities ≥0.75. This percentage corresponds to the precision of the link predictors reported for the PIN.

To compute GO similarities the R package GOSemSim was utilised^43^. Although this package provides different indices to measure similarities between proteins, Wang’s index was used because it was formulated specifically for the GO^44^. GO similarities on the high end of the range [0, 1] are normally good indicators of a potential protein interaction^44^. However, a threshold of 0.544 was preferred in this study, as it corresponds to the upper quartile of all the GO similarities of connected protein pairs in the PIN.

### Hardware used for experiments

All the experiments presented in this paper were executed on a Lenovo ThinkPad 64-bit with 7.7 GB of RAM and an Intel Core i7-4600U CPU @ 2.10 GHz × 4, running Ubuntu 14.04 LTS. The only exception were the link prediction experiments, which were executed on nodes with 100 GB of RAM, within the Mogon computer cluster at Johannes Gutenberg Universität in Mainz.

### Availability

R implementations of the PS model and LaBNE are available at http://www.greg-al.info/code. The network data used in this paper are also available at the same website. The C++ implementation of the fast version of HyperMap used in this paper is available at https://bitbucket.org/dk-lab/2015_code_hypermap.

## Additional Information

**How to cite this article**: Alanis-Lobato, G. *et al*. Efficient embedding of complex networks to hyperbolic space via their Laplacian. *Sci. Rep.* **6**, 30108; doi: 10.1038/srep30108 (2016).

## Supplementary Material

Supplementary Information

## Figures and Tables

**Table 1:**
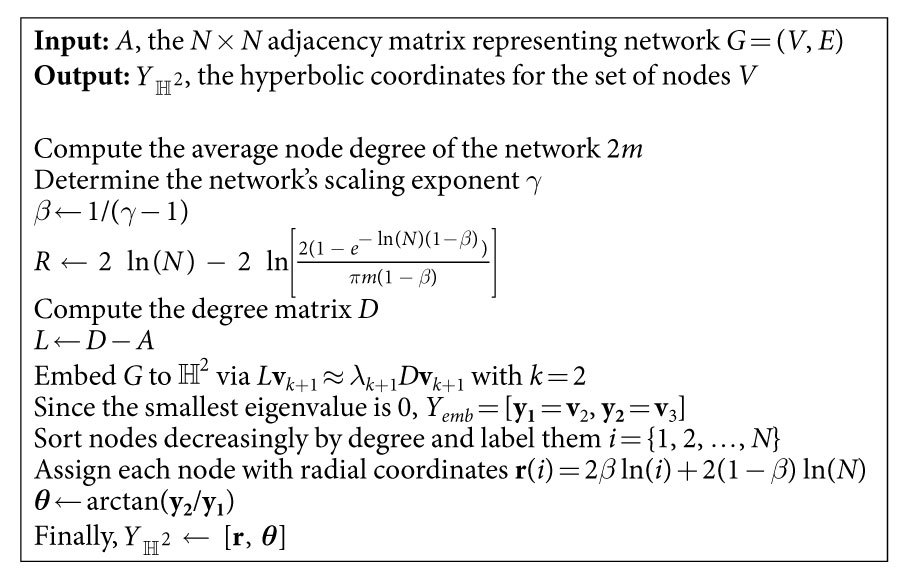
Laplacian-based Network Embedding (LaBNE).

Note that to embed a network *G* to 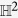, the truncated spectral decomposition of *L* is used. This gives the closest approximation to the eigen-decomposition by a matrix *λ*_*k*+1_ of rank *k *+ 1 and ensures that the computational complexity of LaBNE is *O*(*N*^2^).

**Table 2:** The three real networks analysed in this paper.

Network	*N*	*L*	2*m*	*γ*	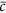	LaBNE (s)	HyperMap (d)
PIN	6906	39303	11.38	2.84	0.20	6.23	0.57
PGP_*t*_	14367	37900	5.28	2.14	0.47	44.67	1.71
PGP_*t*+1_	31524	168559	10.69	2.10	0.38	56.22	5.79
ASI_*t*_	24091	59531	4.94	2.12	0.60	49.73	3.59
ASI_*t*+1_	34320	128839	7.51	2.14	0.71	63.81	6.43

The high quality protein interaction network (PIN), the Pretty-Good-Privacy web of trust (PGP) and the autonomous systems Internet (ASI). The number of nodes *N* and links *L*, average node degree 2*m*, scaling exponent *γ*, clustering coefficient 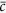, and embedding times in seconds (s) for LaBNE and in days (d) for the most recent and fastest version of HyperMap are reported for each network. For PGP and ASI, the two temporal snapshots used in this paper are also listed. To embed all these networks, HyperMap was fed with the values of *γ* and *m* reported in this table, *T* was set to the values that produce PS artificial networks with the same *N*, 2*m*, *γ* and 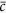 (0.70, 0.33, 0.55, 0.40 and 0.15 respectively) and the algorithm’s speed-up heuristic was applied to nodes with degree *k* < *k*_*speedup*_ = 10. It is worth noting that the performance of HyperMap was reported not to be considerably impacted by the choice of *T*^18^.
