# Supplementary material for: Efficient embedding of complex networks to hyperbolic space via their Laplacian
Source: Sci Rep. 2016 Jul 22;6:30108. doi: 10.1038/srep30108 (PMC4957117; doi:10.1038/srep30108)
Supplement: Supplementary Information [file srep30108-s1.pdf]

SUPPLEMENTARY INFORMATION

Efficient embedding of complex networks to hyperbolic space via their Laplacian

Gregorio Alanis-Lobato*[,1], Pablo Mier[1], and Miguel A. Andrade-Navarro[1]

[1]*Faculty of Biology, Johannes Gutenberg Universität, Institute of Molecular Biology, Ackermannweg 4, 55128 Mainz, Germany*

1 Rationale behind LaBNE

This section provides a detailed description of the rationale behind the Laplacian-based Network Embedding (LaBNE), an approach for the embedding of complex networks to the two-dimensional hyperbolic plane $\mathbb{H}^2$, proposed in the main body of the article that this supplement accompanies. This description is largely based on the justification provided by Belkin and Niyogi for the Laplacian Eigenmaps[1], a manifold learning algorithm for the representation of high-dimensional data in a reduced Euclidean space. Some of the information given in the main body of the article is reproduced here to make this supplement self-contained.

Let us consider only undirected, unweighted, single-component networks, as LaBNE is only applicable to networks with these properties[1]. Moreover, they are assumed to be scale-free (with scaling exponent $\gamma \in [2,3]$) and with clustering coefficient $\bar{c}$ significantly larger than expected by chance. These networks are graphs $G = (V, E)$ with $N = |V|$ nodes and $L = |E|$ edges connecting them. An undirected, unweighted graph can be represented by an $N \times N$ adjacency matrix $A_{i,j} = A_{j,i}\ \forall i, j$, whose entries are 1 if there is an edge between nodes i and j and 0 otherwise. The graph Laplacian is a transformation of A given by $L = D - A$, where D is a matrix with the node degrees on its diagonal and 0 elsewhere.

In the context of manifold learning, most algorithms rely on the construction of a mesh or network over the high-dimensional manifold containing the samples of interest[2,3]. When pairwise distances between samples are computed, they correspond to shortest-paths over the constructed network, allowing for a better preservation of the sample relationships when the data is embedded to low dimensions[1–4]. If there is really a hyperbolic geometry underlying a complex network, it should lie on a hyperbolic plane, with nodes drifting away from the space origin. If the network itself is seen as the mesh that connects samples

*Corresponding author: galanisl@uni-mainz.de

(nodes in this case) that are close to each other[5], it can be used as in manifold learning to recover the hyperbolic coordinates of its nodes. Connected pairs of nodes in the network should be very close to each other in the target, low-dimensional space and, consequently, their angular separation (governed by their similarity dimension according to the Popularity-Similarity model) should also be small. Their popularity or seniority dimension, represented by how far away they are from the space origin, can be easily recovered based on their node degree[5–7].

In particular, embedding of a complex network to the two-dimensional hyperbolic plane $\mathbb{H}^2$, represented by the interior of a Euclidean circle[8], is given by the $N \times 2$ matrix $Y = [\mathbf{y_1}, \mathbf{y_2}]$ where the ith row, Y_i, provides the embedding coordinates of node i. This corresponds to minimising $\frac{1}{2}\sum_{i,j} A_{i,j}||Y_i - Y_j||^2 = \text{tr}(Y^T LY)$, which reduces to $Y_{emb} = \min_{Y^T DY=I} \text{tr}(Y^T LY)$ with D as defined above, I the identity matrix, M^T the transpose of M and $\text{tr}(M)$ the trace of M. Finally, Y_{emb}, the matrix that minimises this objective function, is formed by the two eigenvectors with smallest non-zero eigenvalues that solve the generalised eigenvalue problem $LY = \lambda DY$.

To see that this is indeed the case, let us consider the problem of mapping a network with adjacency matrix A to a line: if $A_{i,j} = 1$ for two nodes i and j (i.e. they are connected), these two nodes should stay as close together as possible on the target line. As a result, we require $y_i \in \mathbb{R}$ that minimise:

$$\frac{1}{2}\sum_{i,j} A_{i,j}(y_i - y_j)^2 \tag{1}$$

Since A is symmetric and $D_{i,i} = \sum_j A_{i,j}$, Equation 1 can be written as:

$$\frac{1}{2}\sum_{i,j} A_{i,j}(y_i^2 + y_j^2 - 2y_i y_j) = \frac{1}{2}\left(\sum_i y_i^2 D_{i,i} + \sum_j y_j^2 D_{j,j} - 2\sum_{i,j} y_i y_j A_{i,j}\right) \tag{2}$$

Note that the two first terms on the right hand side of Equation 2 contribute equally to the expression. As a result, this equation reduces to:

$$\sum_i y_i^2 D_{i,i} - \sum_{i,j} y_i y_j A_{i,j}$$

which in vector form can be written as:

$$\mathbf{y}^T(D - A)\mathbf{y} \tag{3}$$

Based on the definition of the graph Laplacian given above, Equation 3 reduces to:

$$\mathbf{y}^T L\mathbf{y}$$

Therefore, mapping the network of interest to a line consists in finding:

$$\min_{\mathbf{y}^T D\mathbf{y}=1} \mathbf{y}^T L\mathbf{y} \tag{4}$$

where the constraint $\mathbf{y}^T D\mathbf{y} = 1$ removes an arbitrary scaling factor in the embedding[2,3]. Since this is an equality constraint, we can resort to Lagrange multipliers to solve Equation 4:

$$\mathcal{L}(\mathbf{y},\lambda) = \mathbf{y}^T L\mathbf{y} - \lambda(\mathbf{y}^T D\mathbf{y} - 1)$$

Finally, the solution is given by:

$$\begin{aligned}\nabla\mathcal{L}(\mathbf{y},\lambda) &= \left(\frac{\partial\mathcal{L}}{\partial\mathbf{y}}, \frac{\partial\mathcal{L}}{\partial\lambda}\right) \\ &= \left(2L\mathbf{y} - 2\lambda D\mathbf{y}, \mathbf{y}^T D\mathbf{y} + 1\right) = 0\end{aligned} \tag{5}$$

Note that one of the solutions to Equation 5, $\frac{\partial\mathcal{L}}{\partial\mathbf{y}} = 2L\mathbf{y} - 2\lambda D\mathbf{y} = 0$, leads to $L\mathbf{y} = \lambda D\mathbf{y}$. The minimum, non-zero, eigenvalue solution to this generalised eigenvalue problem gives the vector $\mathbf{y}$ that minimises the objective function (Equation 4). More generally, embedding of a network into $\mathbb{R}^d(d > 1)$ is given by the $N \times d$ matrix $Y = [\mathbf{y}_1, \mathbf{y}_2, ..., \mathbf{y}_d]$. Thus, we need to minimise:

$$\frac{1}{2}\sum_{i,j} A_{i,j}||Y_i - Y_j||^2 = \mathrm{tr}(Y^T LY)$$

which reduces to:

$$Y_{emb} = \min_{Y^T DY=I} \mathrm{tr}(Y^T LY)$$

as already discussed above for $d = 2$, which is the focus of this work.

To complete the mapping to the two-dimensional hyperbolic plane $\mathbb{H}^2$, angular node coordinates are obtained via $\theta = \arctan(\mathbf{y_2}/\mathbf{y_1})$ and, as abovementioned, radial coordinates are chosen so as to resemble the rank of each node according to its degree. This is achieved via $r_i = 2\beta\ln(i) + 2(1-\beta)\ln(N)$, where nodes $i = \{1, 2, \ldots, N\}$ are the network nodes sorted decreasingly by degree and $\beta = 1/(\gamma - 1)$ [5,8].

This strategy is valid, because the native representation of $\mathbb{H}^2$, in which the hyperbolic space is contained in a Euclidean disc and Euclidean and hyperbolic distances from the origin are equivalent, is a conformal model. This means that Euclidean angular separations between nodes are also equivalent to hyperbolic ones [8]. On the other hand, the radial arrangement of nodes corresponds to a quasi-uniform distribution of radial coordinates in the disc [8].

References

[1] Mikhail Belkin and Partha Niyogi. Laplacian eigenmaps and spectral techniques for embedding and clustering. *Advances in Neural Information Processing Systems*, 14:585–591, 2001.

[2] Richard S. Zemel and Miguel A. Carreira-Perpiñán. Proximity graphs for clustering and manifold learning. In *Advances in Neural Information Processing Systems*, volume 17, pages 225–232, 2004.

[3] Lawrence Cayton. Algorithms for manifold learning. *UCSD tech report*, CS2008-0923:1–17, June 2005. URL `http://www.lcayton.com/resexam.pdf`. Last visited: 2016-03-30.

[4] J. B. Tenenbaum. A Global Geometric Framework for Nonlinear Dimensionality Reduction. *Science*, 290(5500):2319–2323, December 2000. ISSN 00368075, 10959203. doi: 10.1126/science.290.5500.2319.

[5] Fragkiskos Papadopoulos, Maksim Kitsak, M. Ángeles Serrano, Marián Boguñá, and Dmitri Krioukov. Popularity versus similarity in growing networks. *Nature*, 489(7417):537–540, September 2012. ISSN 0028-0836, 1476-4687. doi: 10.1038/nature11459.

[6] Fragkiskos Papadopoulos, Constantinos Psomas, and Dmitri Krioukov. Network mapping by replaying hyperbolic growth. *IEEE/ACM Transactions on Networking*, 23(1):198–211, February 2015. ISSN 1063-6692, 1558-2566. doi: 10.1109/TNET.2013.2294052.

[7] Fragkiskos Papadopoulos, Rodrigo Aldecoa, and Dmitri Krioukov. Network geometry inference using common neighbors. *Physical Review E*, 92(2), August 2015. ISSN 1539-3755, 1550-2376. doi: 10.1103/PhysRevE.92.022807.

[8] Dmitri Krioukov, Fragkiskos Papadopoulos, Maksim Kitsak, Amin Vahdat, and Marián Boguñá. Hyperbolic geometry of complex networks. *Physical Review E*, 82(3), September 2010. ISSN 1539-3755, 1550-2376. doi: 10.1103/PhysRevE.82.036106.

2 Supplementary figures

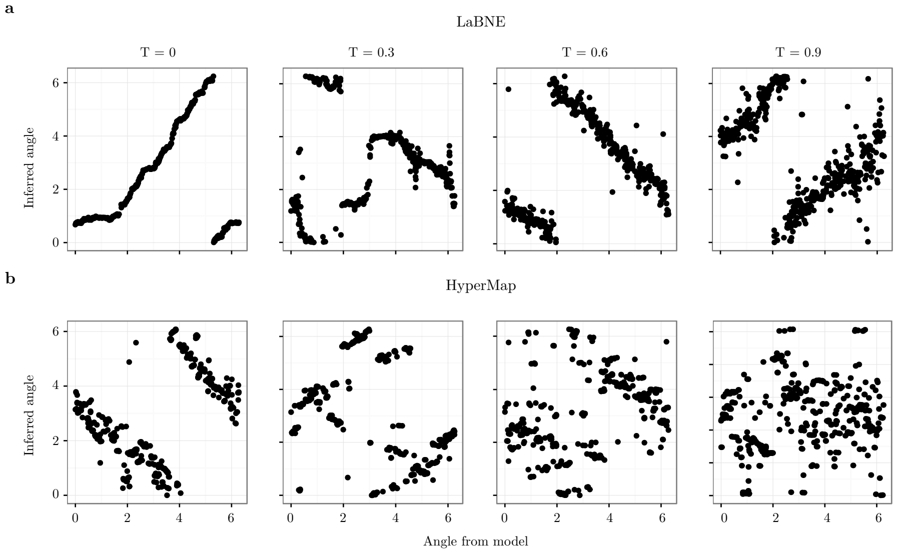

Figure S1: **Real vs inferred angles.** Real vs angles inferred by **(a)** LaBNE and **(b)** the most recent and fastest version of HyperMap for networks with 500 nodes, $\gamma = 2.5$, $2m = 6$ and different temperatures $T = \{0, 0.3, 0.6, 0.9\}$. Axes show the angles in radians.

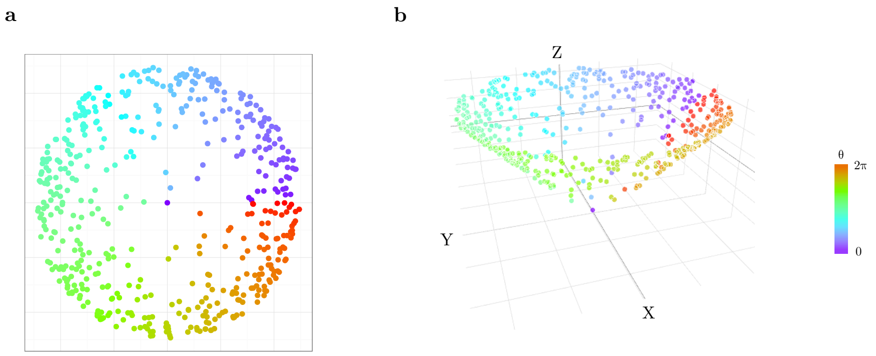

Figure S2: **Network nodes in $\mathbb{H}^2$.** Once a network has been mapped to the two-dimensional hyperbolic plane, its nodes can be visualised in two or three dimensions by means of **(a)** $\mathbb{H}^2$ contained in a Euclidean disc or **(b)** the hyperboloid model of the plane, respectively. The latter case requires the transformation of the polar coordinates found by LaBNE or HyperMap via $(r, \theta) \rightarrow (X, Y, Z) = (r\cos(\theta), r\sin(\theta), \sqrt{r^2+1})$.

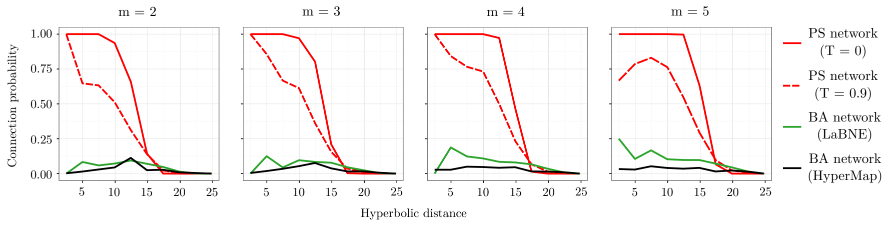

Figure S3: **Mapping Barabasi-Albert networks to $\mathbb{H}^2$.** Barabasi-Albert (BA) networks with 500 nodes and different $m = \{2, 3, 4, 5\}$ were generated and embedded into $\mathbb{H}^2$ using LaBNE and the most recent and fastest version of HyperMap. As already shown by Papadopoulos and colleagues[5], short hyperbolic distances between nodes are not good predictors of link formation in BA networks, mainly due to the fact that their clustering is asymptotically zero[5]. On the other hand, in Popularity-Similarity (PS) networks with the same number of nodes, m and γ as the generated BA networks, but strong clustering ($T = 0$), close nodes are almost always connected. This is in contrast with weakly clustered PS networks ($T = 0.9$), for which the probability that two hyperbolically close nodes connect is lower.
